# Comparison of structures among *Saccharomyces cerevisiae* Grxs proteins

**DOI:** 10.1186/s41021-018-0104-5

**Published:** 2018-09-03

**Authors:** Mohnad Abdalla, Wafa Ali Eltayb, Aadil Yousif

**Affiliations:** 1 Faculty of Science and Technology, Omdurman Islamic University, Omdurman, Sudan; 2 School of Life Sciences, University of Science and Technology of China, Hefei, Anhui 230027 People’s Republic of China; 3 Faculty of Science and Technology, Shendi University, Shendi, Nher Anile Sudan; 4 Faculty of Applied Medical Sciences, University of Tabuk, Tabuk, Saudi Arabia

**Keywords:** Grx, *Saccharomyces cerevisiae*, Protein–protein interactions, Domain, Disorder, Transmembrane

## Abstract

Glutaredoxins (Grxs) comprise a group of glutathione (GSH)-dependent oxidoreductase enzymes that respond to oxidative stress and sustain redox homeostasis. *Saccharomyces cerevisiae* Grx has a similar interaction patterns through its residues between the residues and the environment. The glutaredoxin domain covers 100% of the entire mature Grx1 and Grx8, while the glutaredoxin domain covers ~ 52% of the entire mature Grx6 and Grx7, which have approximately 74 additional amino acids in their N-terminal regions, whereas Grx3 and Grx4 have two functional domains: glutaredoxin and thioredoxin. We have presented the prediction of disordered regions within these protein sequences. Multiple sequence alignment combined with a phylogenetic tree enabled us to specify the key residues contributing to the differences between *Saccharomyces cerevisiae* Grxs and the proportion symmetry.

## Background

The Glutaredoxin family (Grxs) has been portrayed as a part of the thioredoxin-fold oxidoreductase superfamily, which have a catalytic centre with a highly conserved CXXC/S motif. Grxs contribute to various biological functions, including protection against the oxidative damage induced by several reactive oxygen species (ROS) [1], as well as iron-sulfur cluster assembly in reaction to cellular iron availability [2]. Grxs stimulate the reduction of mixed disulfides through low-molecular-weight thiols together with reduced glutathione (GSH) or other proteins in the presence of GSH, NADPH, and glutathione reductase [3]. It has been shown that to lower the pK_a_ value, the N-terminus cysteine of Grxs should be leaving group in the catalytic reaction with a good nucleophile [4, 5]. Grxs can be classified into dithiol and monothiol. Grxs depend on the number of cysteine residues in the active-site of the CXXC/S motif [6–8]. The conserved active-site motifs are usually CPYC in the classical dithiol Grxs and CGFS in the monothiol Grxs. Generally, members of the Grx family are able to bind to GSH through hydrogen bonds in addition to mixed disulfide bonds, as well as electrostatic and highly hydrophobic interactions [9–11].

The kinetic test proposed that Grxs may have two catalytic mechanisms: sequential and ping−pong [12]. The crystal structures of yeast Grx1, Grx2, Grx5, Grx6, and Grx8 have been solved and their enzymatic activities compared. These analyses showed the diversity of Grxs which are encoded by the same organism from both catalytic and structural points of view [13–16].

### Protein–protein interactions network

In a living cell, proteins do not work as single entities, but they form an assortment of functional connections with each other, which are essential in cellular processes. To define how Grxs interact with other host protein families and affect cell functions, the proteins were identified and analysed by searching the STRING database. The STRING database is a tool for the retrieval of functional protein/protein and gene/protein interactions, as well as to determine the probable biological processes affected. The protein interaction network of the Grxs protein was specified using the STRING database [17]. Analysis of protein-protein interactions was performed to identify functionally linked proteins and determine the biological processes that were potentially affected by the known and predicted Grxs interaction partner network. The confidence view screenshot from the STRING database (Search Tool for the Retrieval of Interacting Genes/Proteins) shows the known and predicted protein–protein interaction network of *Saccharomyces cerevisiae* Grx6, which was used as the input for the query. Stronger associations are represented by thicker lines. The required high confidence score was limited to over 75%, and the protein–protein interaction network was limited to the 10 best-scoring hits. The interaction network showed the association between differentially expressed proteins. The interaction map was generated using default settings (high confidence of 0.7 and 7 criteria for linkage: neighbourhood, gene fusion, co-occurrence, co-expression, experimental evidence, existing databases and text mining). Based on its literature value and database, it created the interactions with the protein of interest. Using the STRING database to create protein interactions is very helpful to understand the protein because it shows which proteins interact with Grx6.

**Fig. 1:**
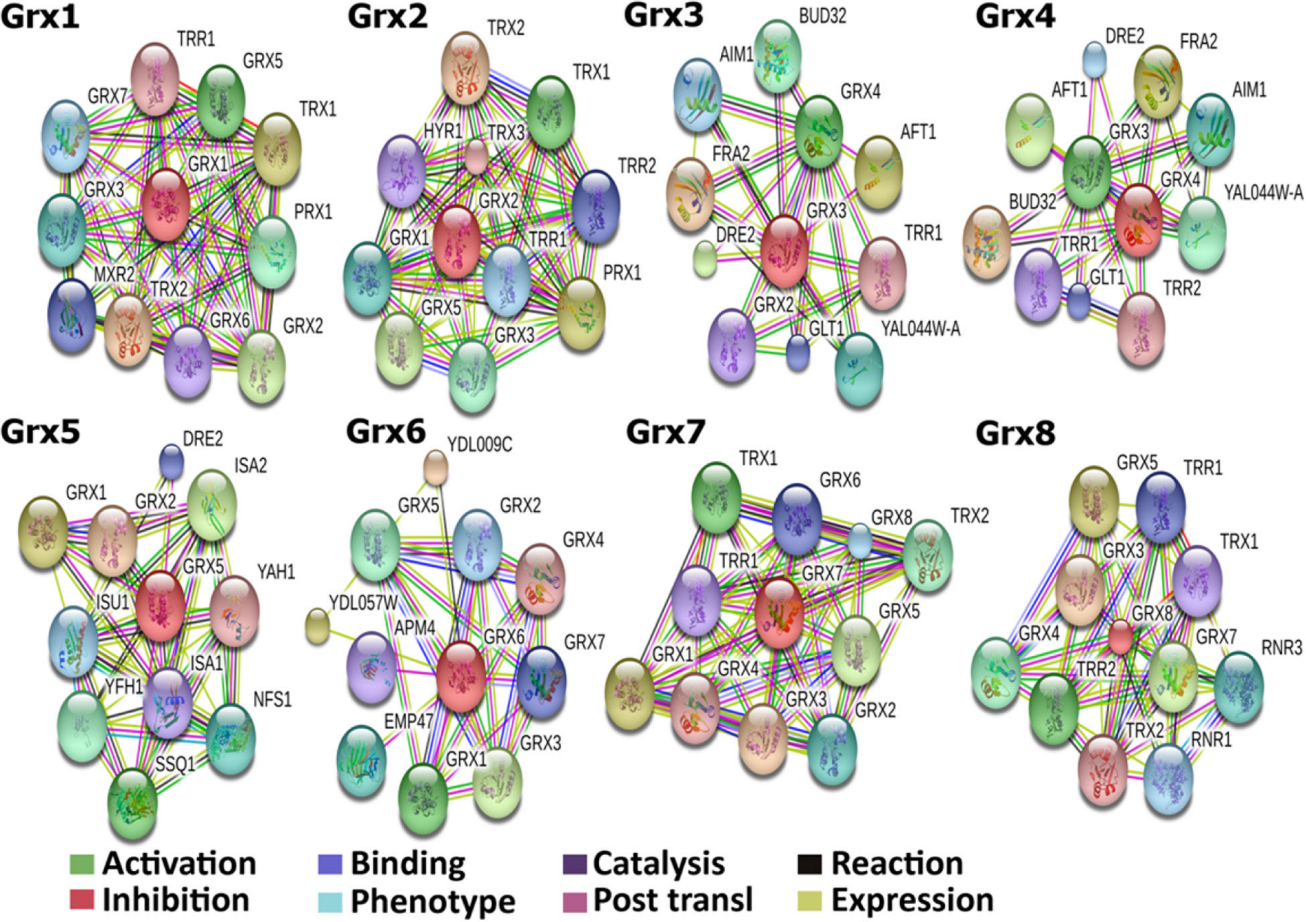
Protein-protein networks constructed by the STRING analysis [17]. Modes of action are shown in different colours. *Saccharomyces cerevisiae* Grx protein, UniProt ID: Grx1: P25373; Grx2: P17695; Grx3: Q03835; Grx4: P32642; Grx5: Q02784; Grx6: Q12438; Grx7: P38068; and Grx8: Q05926

The *Saccharomyces cerevisiae* Grxs interaction network shows that Grxs proteins form a highly complex, exceedingly organized network of interacting proteins as expected, as shown by the search tool for the Grxs of interacting proteins and/or genes (STRING) network analysis. Most identified proteins show main functional involvement. Clusters which contain thioredoxin isoenzyme (TRX1 and TRX2 and TRX3) [18], thioredoxin reductase (TRR1), methionine-R-sulfoxide reductase (MXR2), mitochondrial peroxiredoxin (PRX1), thiol peroxidase (HYR1), NAD(+)-dependent glutamate synthase (GLT1) [19, 20], and protein kinase (BUD32) are involved in the negative regulation of the transcription of iron regulon (FRA2) and proteins of unknown function (YAL044W-A, YDL009C, YDL057W). Activator of ferrous transport (AFT1) is a transcription factor involved in iron utilization and homeostasis [21]. FRA2 is a protein involved in the negative regulation of transcription of iron regulon [22, 23]. BolA-like protein (AIM1) is a protein involved in mitochondrial function and organization. Until now literature review showed that both Grx3 and Grx4 interacted with AFT1 to form hetero-oligomers [24]. However, there has been no information made available about an additional interaction. Analysis of the activity and expression of these directly associating proteins in *Saccharomyces cerevisiae* may provide insights and thus is a good avenue for future study (Fig. 1).

### Grx domain

**Fig. 2:**
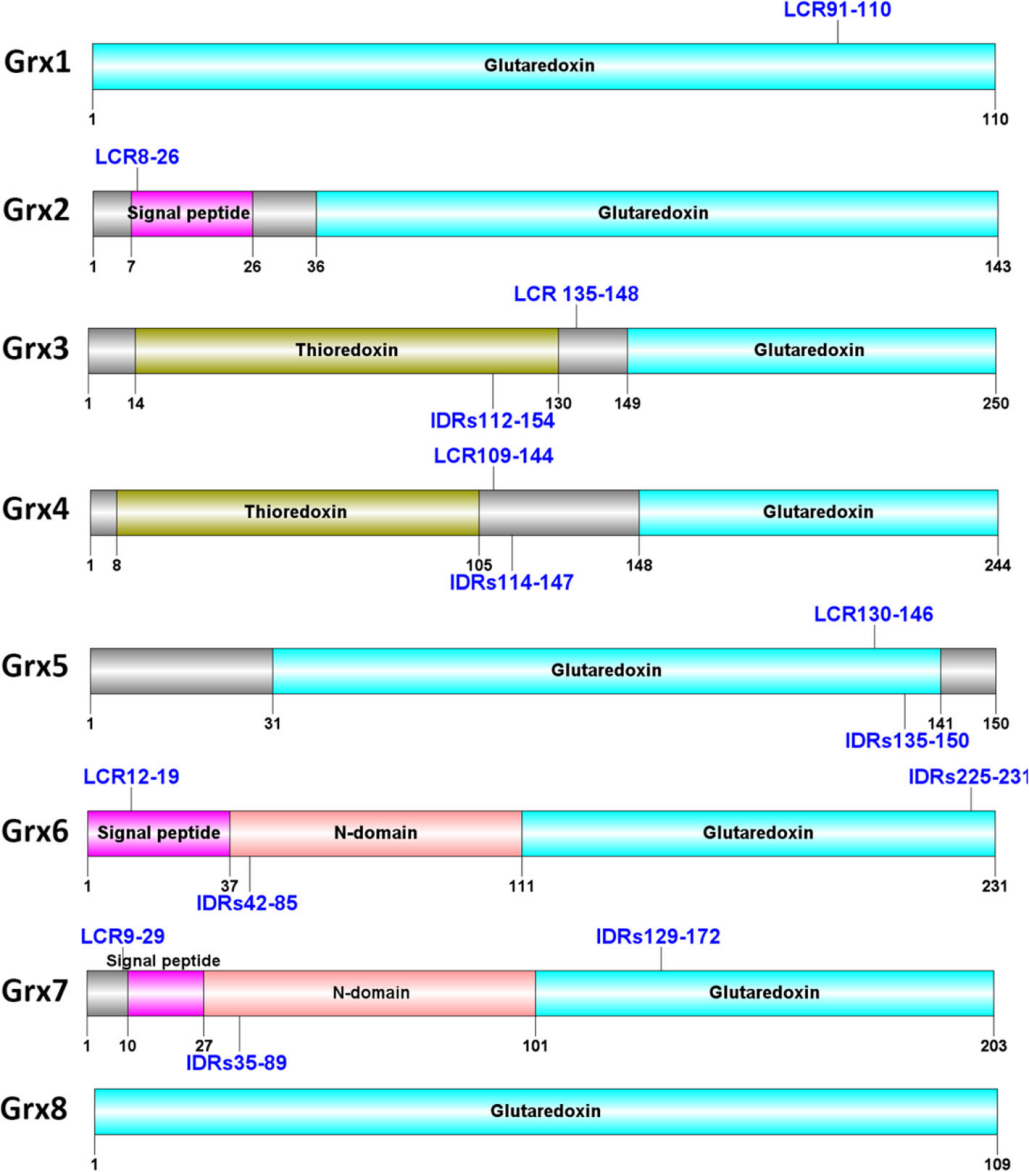
Grxs domain. Intrinsically disordered regions (IDRs); low complexity regions (LCR)

Grx is a small, single or multi-domain (Fig. 2) protein. Analysis of the protein domain organization showed that the glutaredoxin domain covers 100% of the entire mature Grx1 [14] and Grx8 [15], while the glutaredoxin domain covers ~ 52% of the entire mature Grx6 [6] and Grx7, which have an additional approximately 74 amino acids in their N-terminal regions. In contrast, Grx3 and Grx4 have two functional domains, glutaredoxin and thioredoxin. Glutaredoxin represents a proportion of ~ 40%, while thioredoxin represents a proportion of ~ 46% of the entire mature protein, Grx2 glutaredoxin represents the proportions of ~ 75% [14] and Grx5 glutaredoxin represents the proportions of ~ 68%. Low-complexity regions (LCRs) are amino acid sequences that contain repeats of single amino acids or short amino acid motifs.

### Grx disorder

**Fig. 3:**
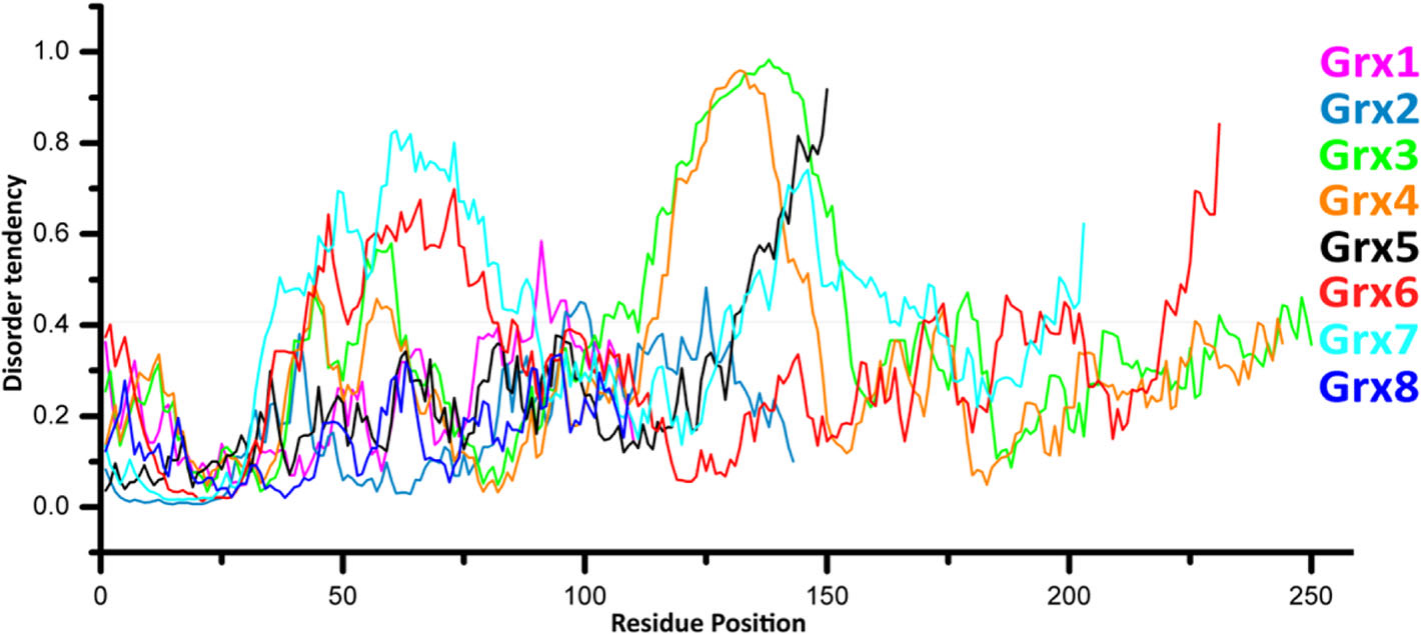
Disorder profiles of Grx 1–8 sequences. Values above 0.4 are regarded as disordered. The data was obtained from the IUPred2A [25]

Grxs also contain disordered regions, while they have structured regions. However, only Grx7 is almost fully disordered in its native state. From the disorder prediction we can see that the large disordered region in Grx3 is between residues 112 and 154, in Grx4 it is between residues 114 and 147, in Grx5 it is between 130 and 146, and there are two disordered regions in Grx6 between residues 42 and 85 and between residues 225 and 231. Grx7 also has two disordered regions, between 35 and 89 as well as between 129 and 172. The Grx7 sequences consist of many random coil conformations with only a small ratio of helices and sheets. The large disordered region is located in the C domain in Grx3 and Grx4, while it is located in the N domain in Grx6, and in Grx7 the disordered regions are located at both the N and C domains. In fact, the large disordered region loops with a high degree of mobility. Because of this, until now we were not able to determine the three-dimensional folded structures of Grx3, Grx4, Grx7, and the N domain of Grx6. Grx1, Grx2, Grx5, Grx6 C domain and Grx8 do not contain a large disordered region. The *Saccharomyces cerevisiae* Grxs are classified to three categories: structured, structured but containing a disordered region, and almost fully disordered. In addition, Grxs contain low complexity regions: Grx1 91–110, Grx2 8–26, Grx3 135–148, Grx4 109–144, Grx5 130–146, Grx6 12–19 and Grx7 9–29. Numerous disordered regions imply low complexity sequences, and although low complexity sequences are a strong sign of disorder (Fig. 2), the inverse is not necessarily correct. Many LCRs are highly unstable due to replication slippage and recombination. However, not all disordered regions have low complexity sequences. Disordered regions have a low utility for the prediction of secondary structure. Sometimes, Grx sequences sustain a transformation from disorder to order upon interacting with ligands (GSH), such as the small disordered region in Grx6 225 to 231 (Fig. 3). The data was obtained from the IUPred2A [25].

### Grx superimposed

Five structures are available among the eight solved to date. In general, the 3D structures of the Grxs superfamily are quite similar. Similarity between the structures is seen in *Saccharomyces cerevisiae* Grxs with 4 beta strands, except Grx6 with 6 beta strands. To assess how Grxs structural diversity, a 3D structure alignment was performed by the PyMOL 2.1 [26].

Grx6-3l4n consists of 6α helices and 6β strands, while Grx6-5j3r consists of 5α helices and 6β strands. That means the monomer has one more α helix, with the extra α helix situated between β2 and α4. Leu125 and Ser126, which are part of α1 in 5j3r, are located in the loop between α1 and β1 in 3l4n. The RMSD (root mean square deviation) between Grx6-5j3r and Grx6-3l4n is 0.37 Å.

There are slight conformational changes in Grx2-3ctg and Grx2-3ctf at the active site as well as conformational rearrangements between the residues Thr59 and Lys65. However, the major conformational change is 10.91 Å at Tyr60 when it is bound to GSH [14]. The RMSD between Grx2-3ctg and Grx2-3ctf is 0.3 Å. Grx2-3ctg Leu138-Gln143 are located at the end of the C-terminus as a loop, but in 3ctf this amino acid it is part of α6. Gln50 and leu51 are located in the loop between α1 and β1 in 3ctf, while there is a part of α1 in 3ctg. Grx2-3ctg and Grx2-3ctf consist of 6α and 4β.

**Fig. 4:**
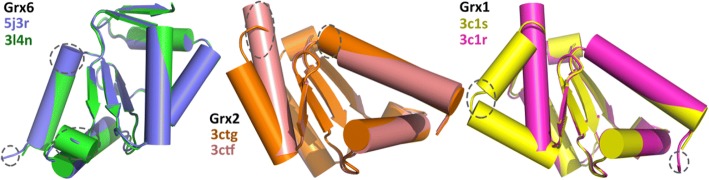
Superimposed 3D models of Grx6 (5j3r-3l4n), Grx2 (3ctg-3ctf) and Grx1 (3cts-3c1r) showing conformational changes. The models were created by the PyMOL 2.1 [26]

There are dramatic conformational changes in Grx1-3c1s and Grx1-3c1r at the active site as well as conformational rearrangements in Thr25 and Tyr26. However, the major conformational change is 12.22 Å at Tyr26 in the glutathionylated form. Gly98 in Grx1-3c1s is located in the loop between α6 and α7, while it is located in α5 at 3c1r. In addition, α6 in Grx1-3c1r is longer than α7 in Grx1-3c1s, α6 in Grx1-3c1r between Gly98 and Ala109, and α7 in Grx1-3c1s between Glu99 and Glu105. This means that conformational changes of Grx depend on the type of ligand. Grx1-3c1s is an amino acid involved in GSH-forming hydrogen bonds with (Asp89-Asn88-Cys27-Val75-Lys24-Gln63). These interactions are also determined in other Grx complexes with GSH, while two water molecule-mediated hydrogen bonds exist between (Asp89-Asn88) and MES in Grx1-3c1r. The RMSD between Grx1-3c1s and Grx1-3c1r RMSD is 0.51 Å. Grx1-3c1s consists of 7α helices and 4β strands, while Grx1-3c1r consists of 6α helices and 4β strands (Fig. 4).

Grx proteins are constantly changing their conformation in both their oxidized and reduced forms. It is not clear whether these slight differences have an important impact on substrate selectivity or protein function.

### Grx transmembrane

**Fig. 5:**
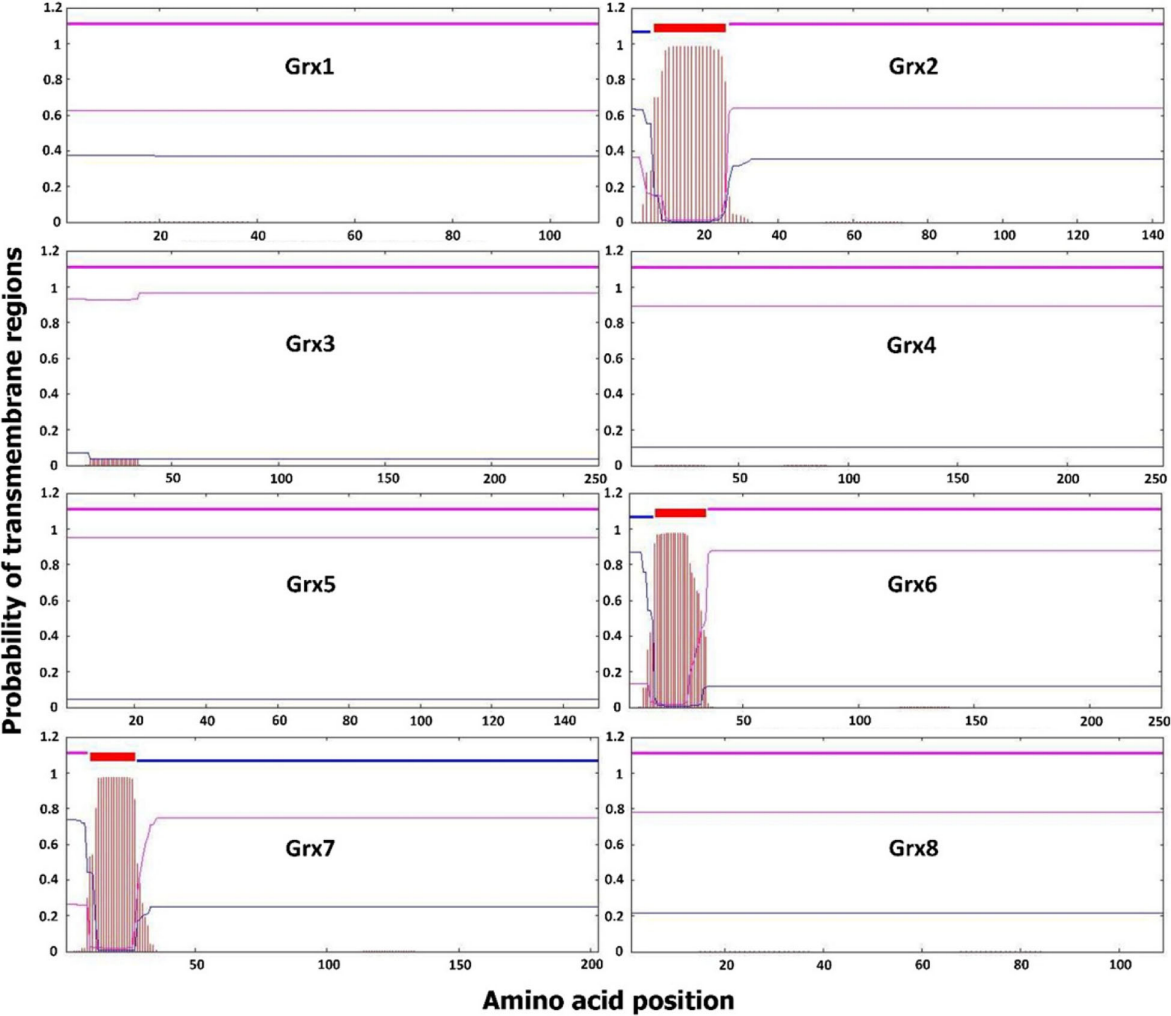
Prediction of transmembrane regions of Grxs. Transmembrane regions are shown in red, and other regions are predicted to be either outside (pink) or inside (blue) the membrane. Data was obtained from the TMHMM Server v. 2.0 [27]. X-axis refers to amino acid position; while Y-axis refers to probability of transmembrane regions. Pink line refers to intracellular proteins will blue line refers to extracellular proteins

Single protein regions are known to have various types of functions. One of these functions leads to the translocation of the polypeptide into the endoplasmic reticulum, which is very important to identify. We therefore analysed the transmembrane regions [27]. Grxs transmembrane regions were found only in Grx2, Grx6 [28], and Grx7. Signal peptide prediction showed only one signal peptide in each of them, and all of them localized at the N-terminus (Fig. 5). All *Saccharomyces cerevisiae* Grxs are predicted as intracellular proteins except that Grx7 was predicted as extracellular protein (Fig. 5). The existence of a signal sequence and a disorder sequence also backs up this localization. Additionally, this localization has not been demonstrated before and gives us extra details about the of *Saccharomyces cerevisiae* cell wall architecture.

### Grxs multiple sequence alignment

**Fig. 6:**
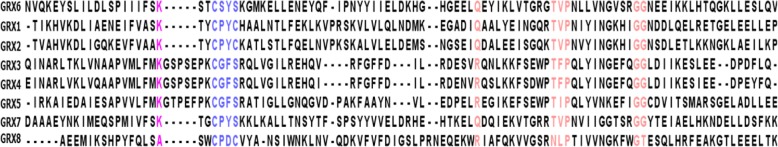
*Saccharomyces cerevisiae* Grxs multiple sequence alignment. The alignment was created by the Clustal W [29]

Highlighted are a number of structural similarities and conserved motifs that are common to plants, animals, humans, and bacteria. However, one or two differences may affect protein function. The deduced amino acid sequences of Grx comprised of a conserved TVP and GG at the C domain involves GSH binding. TVP and GG are the second most commonly matched sequence in the Grxs superfamily, after the active site (Fig. 6). However, despite the sequence similarity, there are some crucial differences in the active site architectures. The one cysteine residue is extremely well conserved, and the Grxs active sites have similar biochemical properties. Information about the biochemical and/or biological properties is shown in Table 1.

**Table 1:** Characteristic comparison among the *Saccharomyces cerevisiae* Grxs proteins

	YCL035C\GRX1	YDR513W\GRX2	YDR098C\GRX3	YER174C\GRX4	YPL059W\GRX5	YDL010W\GRX6	YBR014C\GRX7	YLR364 W\GRX8
Number of amino acids	110	143	250	244	150	231	203	109
Formula	C_549_H_886_N_146_O_170_S_4_	C_715_H_1161_N_181_O_213_S_5_	C_1250_H_1922_N_322_O_411_S_7_	C_1226_H_1910_N_316_O_389_S_6_	C_762_H_1200_N_196_O_227_S_6_	C_1139_H_1834_N_306_O_361_S_6_	C_991_H_1564_N_274_O_320_S_4_	C_571_H_879_N_149_O_160_S_4_
Molecular weight(Da)	12,380.19	15,861.47	28,261.34	27,492.90	16,931.45	25,783.28	22,565.20	12,519.40
pI	4.98	6.73	4.37	4.57	4.85	6.01	5.64	7.78
Instability index	40.59	32.24	51.79	46.11	60.33	35.77	41.82	35.73
Aliphatic index	107.27	104.97	81.12	83.93	86.53	89.87	81.58	83.12
Grand average of hydropathicity (GRAVY)	−0.248	0.024	−0.470	−0.355	−0.259	−0.412	−0.437	−0.260
Number of cysteines	2	2	3	2	2	1	1	2
PDB ID	3C1R 3C1S	3CTF 3CTG	5Y4U 5Y4T		3GX8	5J3R 3L4N		2 M80
Ligands	MES GSH				SO4	GSH + Fe2 S2 GSH		
location	Cytosolic	Major one at the cytosol and a minor one at mitochondria	Nucleus	Nucleus	Mitochondrial matrix	Vacuole	Membrane	Cytoplasm
Active site	CPYC	CPYC	CGFS	CGFS	CSYS	CSYS	CPYS	CPDS
Function	In defence against oxidative stress	In defence against oxidative stress	Regulate the activity of transcription factors such as Aft1	Regulate the activity of transcription factors such as Aft1	Participates in the synthesis of iron–sulfur clusters.	The First Monothiol Grxs with Activity in the HEDS Assay and first Monothiol Grxs Forming Noncovalently Linked Dimers.		Not an essential gene
Iron-Sulfur Cluster Binding	No	No	Yes	Yes	Yes	Yes	No	No
HEDS assay	Yes	Yes	No	No	No	Yes	Yes	Very weak

The key modifications in Grxs are at the active-site CXXC motif and at the GSH-recognition site. Grx8 utilizes a CPDC motif instead of the CPY(F)C motif found in other Grxs. Moreover, the TVP motif in almost all Grxs is involved in the bond with the cysteine moiety of GSH. The Gln residue is implicated in stabilizing the glycine moiety of GSH.

### The phylogeny of Grx

**Fig. 7:**
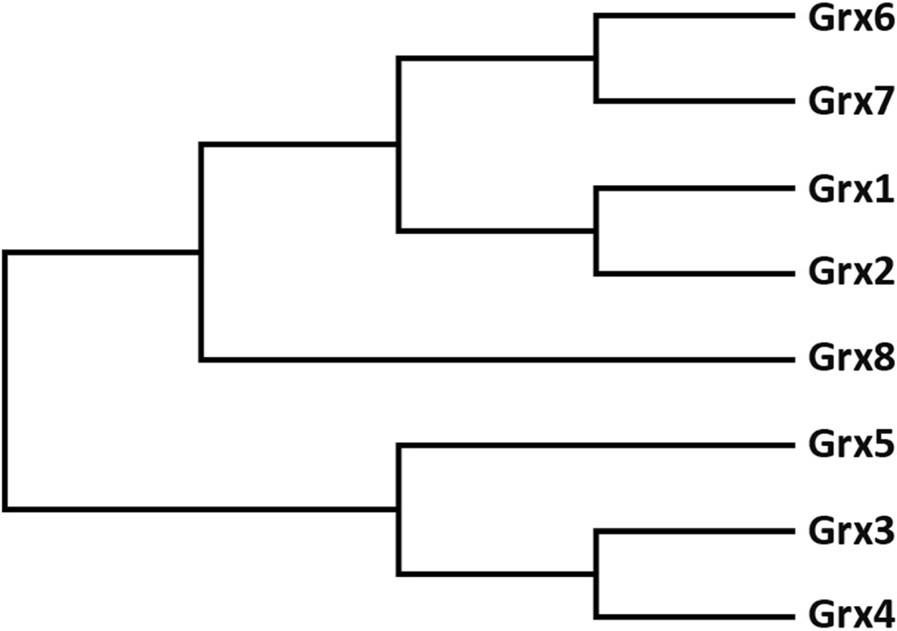
Phylogenetic dendrogram of *Saccharomyces cerevisiae* Grxs. The phylogenetic tree derived from the Clustul W multiple sequence alignment was constructed by the maximum parsimony method of the MEGA7 software [30]

The yeast *Saccharomyces cerevisiae encodes* ten Grxs, eight of which have been characterized, including five monothiol Grxs and three dithiol Grxs. There is a high degree of similarity shared between the Grx1 and Grx2 sequences (64%), the Grx3 and Grx4 sequences (66.8%), and the Grx6 and Grx7 sequences (37.5%). A phylogenetic tree (Fig. 7) which was constructed for the validated *Saccharomyces cerevisiae* Grxs reveals that Grx1 and Grx2, Grx3 and Grx4, and Grx6 and Grx7 well-defined to be more closely related to each other than to Grx5 and Grx8. However, there are some differences among Grx1, Grx2, Grx6 and Grx7 in GSH-dependent oxidoreductase activity because of their slightly different in the active-site structures. Closely related pairs include Grx1 and Grx2, Grx3 and Grx4, and Grx6 and Grx7.

### Grx protein expression levels

**Fig. 8:**
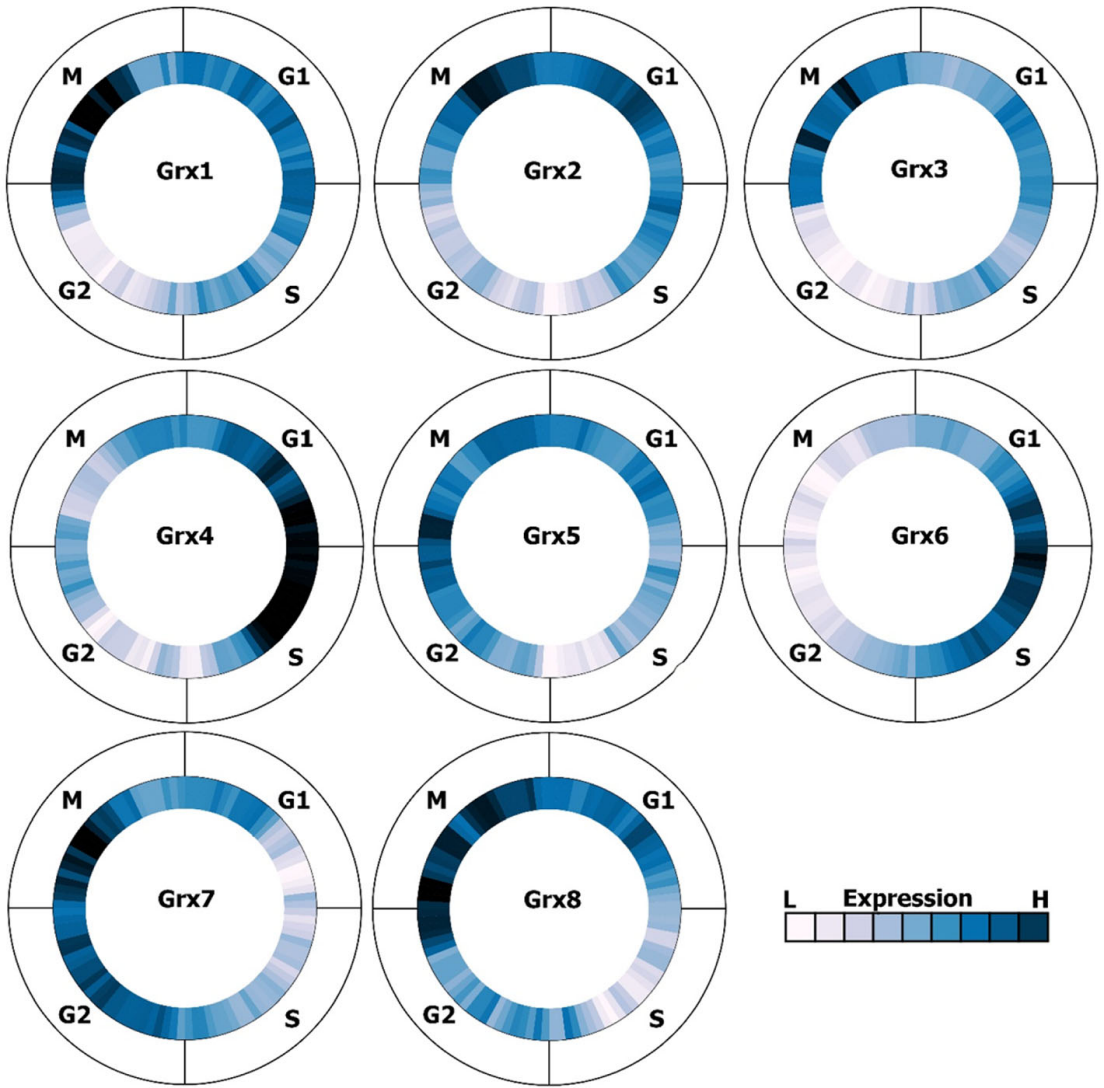
Grxs1–8 protein expression levels in a four-stage model of the cell cycle. The figures were created by the Cyclebase [31]

The prediction shows that Grx4 possesses the highest level of the expression and longest period of expression compared to other Grxs. Grx6 expression stage is almost opposite to Grx7. Grx6 increases its level of expression between the end of stage G1 and the middle stage of S, while Grx7 increases its level of expression between the middle stage of stage G2 and the middle stage of M. However, Grx1 and Grx2 have very low expression in the G2 stage, while the expression increases for both of them in stage M. Furthermore, Grx3 increases its level of expression in stage M, while Grx4 possesses a low level of expression in the G1 stage (Fig. 8).

## Conclusion

Due to the sequence conservation, overlapping protein interactions, and matching of disordered profile, glutaredoxin domain of a similar length in eight proteins leads to similar functions. It is clear from this analysis that a considerable number of amino acid sequences represent a random coil configuration in Grx7, leading to an inability to solve the structure. Eight proteins have almost same interaction partner as the Grx protein with high similarly and conservation.

## Abbreviations

Cys: Cysteine
ER: Endoplasmic reticulum
Fe/S: Iron-Sulfur
Grx: Glutaredoxin
GSH: Glutathione
PDB: Protein data bank
